# Moral leniency towards belief-consistent disinformation may help explain its spread on social media

**DOI:** 10.1371/journal.pone.0281777

**Published:** 2023-03-22

**Authors:** Laura Joyner, Tom Buchanan, Orkun Yetkili

**Affiliations:** School of Social Sciences, University of Westminster, London, United Kingdom; Roma Tre University: Universita degli Studi Roma Tre, ITALY

## Abstract

The spread of false and misleading information on social media is largely dependent on human action. Understanding the factors that lead social media users to amplify (or indeed intervene in) the spread of this content is an ongoing challenge. Prior research suggests that users are not only more likely to interact with misinformation that supports their ideology or their political beliefs, they may also feel it is more acceptable to spread. However, less is known about the influence of newer, issue-specific beliefs. Two online studies explored the relationship between the degree of belief-consistency of disinformation on users’ moral judgements and intentions to spread disinformation further. Four disinformation narratives were presented: disinformation that supported or undermined the UK Government’s handling of COVID-19, and disinformation that minimised or maximised the perceived risk of COVID-19. A novel scale for measuring intentions to contribute to the spread of social media content was also used in study 2. Participants reported greater likelihood of spreading false material that was consistent with their beliefs. More lenient moral judgements related to the degree of belief-consistency with disinformation, even when participants were aware the material was false or misleading. These moral judgements partially mediated the relationship between belief-consistency of content and intentions to spread it further on social media. While people are concerned about the spread of disinformation generally, they may evaluate belief-consistent disinformation differently from others in a way that permits them to spread it further. As social media platforms prioritise the ordering of feeds based on personal relevance, there is a risk that users could be being presented with disinformation that they are more tolerant of.

## Introduction

Social media platforms have become a ubiquitous part of everyday life in industrialized societies. They are widely used for communication, and for sharing information of all kinds. Unfortunately, not all of that information is true. Around the world, online misinformation is seen as a significant cause for concern, with more than half of internet users worrying about encountering false material [1]. Policymakers share this concern, characterising it as a significant threat to democracy [2]. Yet, research also suggests that people at both ends of the political spectrum may associate the spread of misinformation with opposing political beliefs [3]. Factors such as perceptions of accuracy [4,5], and consistency with political affiliation [6] are thought to influence moral evaluations of misinformation. In turn, it has also been suggested that said moral adjustments may influence user interactions with misinformation [4,7]. Given the important role of perceived morality in self-regulation [8], any belief-related leniency in moral evaluations of misinformation may help explain why users go on to contribute to its digital spread.

The present research examines whether issue-specific beliefs play a role in how misinformation and disinformation are evaluated and spread within social media contexts. “Disinformation” is a term arising from studies of political communication. It is used to refer to false material that is potentially harmful and is shared with harmful intent [9]. This is related to, but separate from, “misinformation” which is false information that is shared inadvertently. Day-to-day, social media users may interact with content based on relevance [10–12] and in turn these ‘signals’ allow platforms to serve users with potentially relevant content. Some disseminators of disinformation have been known to take advantage of these factors, utilising strategies such as microtargeting of advertisements and community development to target users with curated messaging [13]. Disinformation campaigns have previously targeted a range of audiences, including underrepresented groups [13]. They have also targeted pre-existing tensions, including divisions inside political parties [14]. In these instances, ideology or partisanship may not best explain differences in user-interactions. The technological and strategic realities therefore indicate that social media related misinformation research may require more granular approaches. In response to this need, the two studies presented here investigate the influence of degree of belief-consistency with misinformation on participants’ spread intentions and moral evaluations.

Importantly for the purposes of this paper, the same piece of false information can be considered as either disinformation or misinformation depending on who is sharing it, and why. If I tell you a lie, for malicious reasons, that might be disinformation. If you believe it and pass it on to your friends, that would be misinformation. For purposes of clarity, we will use the term “misinformation” where participants are interacting with false material that they have no reason to believe is untrue, and “disinformation” where participants are interacting with false material that they have been told is not true.

Much of the spread of false material online can be attributed to the actions of individual social media users [15]. This spread may be conscious and deliberate when people share it to their own social networks. People may also share material simply by interacting with it: engaging with content in ways such as ‘liking’ it causes social media platforms to show it to a greater number of people, expanding its reach through algorithmic propagation [16]. Why do people do this? A key factor in whether people choose to interact with false material online may be whether or not it is consistent with their beliefs.

### Belief-consistency and the spread of disinformation

Social media users may interact with content they encounter online—which may actually be misinformation—to express opinions or beliefs [17]. For instance, users are more likely to interact with or believe misinformation that is consistent with their ideology or ‘political beliefs’ [18–20]. However, rather than representing specific beliefs, research in this area has tended to measure ‘political belief’ using categorical indicators of American partisanship [4] or self-reported placement along a political orientation scale [18–20]. While these findings are indeed valuable in developing an understanding of why people digitally spread misinformation, there are also potential limitations to using broad categorisations. Arguably, it is not assumed that supporters of a single party hold the same beliefs, nor do people interact with online content simply because of broad ideological appeal. Indeed, analysis of real-life interactions with disinformation has previously found that applying broad-ideological categories may hide potentially important patterns of interaction [21]. Specifically, Freelon et al. found that when inauthentic accounts that are thought to have been designed to target specific groups (in this case, accounts seeking to mimic Black activists) are grouped within a more general ideological category for analysis, it can hide potentially important indicators of increased interactions [21]. Arguably, given the strategic nature of disinformation dissemination on social media [13], it may therefore be valuable to take said strategies into account when seeking to understand why users contribute its spread.

With this in mind, one approach has been to use attitudes as a predictor of misinformation susceptibility. For instance, previous studies have found negative attitudes towards immigration may be more likely to interact with [22] and believe [23] immigration-related misinformation. One explanation for this may be because people prefer information which confirms stereotypes [24]. It may also relate to a tendency to interact with misinformation that induces emotions [15]. However, these studies also found levels of attitude consistency against other narratives, suggesting another factor may relate to the misinformation message itself. Indeed, others have found that an individuals’ position on issues such as reproductive health rights may predict interactions with misinformation that supports said position [25,26]. People are therefore more likely to interact with misinformation that is consistent with their attitudes.

There are of course arguments that certain individuals may be potentially more susceptible than others to misinformation, for instance, people who hold certain beliefs about the world. Research has found that specific beliefs, such as those around science, may predict misinformation sharing generally [27], while lower trust in scientists has also been linked to increased acceptance of COVID-19 misinformation [28]. However, as the previously discussed research on attitude-consistency demonstrates [25,26], the selection of misinformation presented to participants may influence the direction of results. It therefore becomes difficult to ascertain whether distrust in scientists is truly a predictor of increased misinformation susceptibility, or if the presented misinformation simply appealed to such beliefs (in this instance, misinformation statements presented to participants in this study were either conspiratorial in nature (e.g. COVID-19 was created in a lab) or referred to faux home-treatments and tests [28]). In reality, ‘COVID-19 misinformation’ constitutes a much wider spectrum of narratives, with previous work also finding that individual’s susceptibility to COVID-19 misinformation differed across narratives [29]. Additionally, as high trust in scientists may predict belief in and intentions to share virus pseudoscience [30], there may also be situations where low trust in scientists leads to users being less likely to share than others. This further illustrates the need to develop a better understanding of how methodological decisions may influence outcomes in misinformation research. To date, a small number of studies have distinguished between different types of misinformation to better explain susceptibility [23] and intentions to interact [22], but no studies yet have considered how misinformation themes made up of opposing sentiments could influence relationships between beliefs and spread.

Furthermore, the majority of research looking at intentions to spread misinformation has focused on established, more stable beliefs (such as attitudes towards abortion [25,26]) or ideology generally. Research focusing on more recently established or less stable beliefs is, however, limited. Yet, times of crisis such as the COVID-19 pandemic are examples of situations where beliefs that had not previously existed can quickly become relevant. Unlike more established beliefs, these beliefs may fluctuate over time. For instance, in the context of the pandemic, levels of public trust in how the UK government handled COVID-19 was impacted by major events such as the calling of the first lockdown and political scandals [31]. As the targeted dissemination of disinformation can occur during times of crisis, it is increasingly important to understand how these less stable, issue-specific beliefs may influence user-interactions with misinformation.

Notably, beliefs can be thought of as probability assessments of a particular outcome being true [32]. This ties in another reason social media users may be more likely to spread misinformation: because they see it as accurate or believable [20,25,26]. Where beliefs represent what a person perceives to be ‘true’ [33], belief-consistent content may also feel ‘true’ in a way that makes users more likely to interact with it. From this perspective, the closer a narrative aligns with their beliefs, the more likely it is that it may be viewed as accurate. For instance, material that is consistent with our attitudes may also be judged as more accurate [34], plausible [35] and credible [23]. Furthermore, research has also suggested when information known to be false has a narrative which feels broadly true, people may be more likely to interact with it [4,36]. Belief-consistent disinformation and misinformation may therefore be evaluated differently to general ‘disinformation’, and that difference may help to explain why some users spread it.

Furthermore, while social media users may care about the accuracy of the content they spread [37], their perceptions of accuracy may not always be objective. People can interpret information in a way that allows them to confirm their existing beliefs [38,39]. Additionally, while it has previously been argued that engaging in deliberative reasoning will help people accurately identify false content in an un-biased manner [40], from a motivated reasoning perspective, these accuracy judgements of misinformation could involve conscious or unconscious cognitive strategies related to a person’s goals [41]. In some instances, these goals would be related to the veracity of information (e.g. its objective truthfulness). However, as goals can also reflect the achievement of desired, directional outcomes, ‘accuracy’ goals can also be subjective [42]. If that is the case, then subjective interpretations of accuracy may allow social media users to evaluate belief-consistent content as ‘accurate’. This could even extend to belief-consistent content known to be factually inaccurate if the underlying message is seen as broadly ‘true’. For instance, supporters of President Trump previously described a need to take his statements seriously rather than literally [43]. The closer that disinformation matches a person’s perception of ‘truth’, the more likely they may feel the sentiments are justified, regardless of underlying veracity.

### Moral cognition and disinformation spread

People generally want to be perceived as being ‘moral’, and so behaviour may be self-regulated in line with moral standards and moral norms, allowing an individual to conduct themselves in a way that is desirable to both the self and others [44]. Arguably, spreading disinformation is one type of behaviour that would normally be considered undesirable. Research to date generally supports this, showing users may avoid spreading disinformation due to reputational concerns [45] and are less likely to interact with disinformation that they perceive to be ‘immoral’ [7]. However, given the scale and pace of social media platforms, it may be unlikely that users deliberate about the ethics of every item they encounter when online. Instead, they may rely on automatic “affective flashes” to provide signals of potential moral violations [46]. Encountering a piece of content that presents clearly as disinformation (for example, an implausible story featuring a ‘fact check’ label) may produce a sense of ‘wrongness’ that leads users to refrain from interacting with it. Content that conflicts with strongly held beliefs may also do the same.

Yet, false or misleading content may not always represent a clear moral violation to social media users. This may be particularly relevant for belief-consistent misinformation, where information presented could be consistent with what a person believes to be ‘true’ even though it is factually incorrect. Unless it is known by the user to be disinformation, they may not readily perceive a piece of belief-consistent content as something that could potentially deceive others. Notably, people who hold inaccurate beliefs and spread false but belief-consistent information are not, by definition, acting ‘dishonestly’ due to their lack of intention to mislead [47]. From a user perspective, spreading belief-consistent ‘content’ (that happens to be misinformation) may not present a moral violation of any truth or honesty related norms. Without a sense of ‘wrongness’ to guide self-regulation, user’s intentions to interact with belief-consistent misinformation may be no different to interactions with similar truthful content.

However, some people will spread disinformation with the knowledge that the content is false or misleading. While there may be people who do not feel that the action of spreading disinformation is wrong generally, others may be making selective exceptions for this behaviour, perhaps by prioritising different norms or values. For instance, when an attitude is moralised it can lead to more favourable evaluations of sources of attitude-consistent information, regardless of actual and perceived credibility [6]. That does not mean that these individuals would be less tolerant of dishonesty than others generally, but suggests such lenience is context specific. To avoid the negative personal impact of violating personally-important moral values, an otherwise ‘immoral’ act (in this instance, dishonesty) may be cognitively reconceptualised [48]. For instance, people are less likely to label pro-social acts of ‘dishonesty’ as ‘lies’ [49]. As ‘justified’ moral violations may even be emotionally beneficial rather than detrimental [50] this could impact behavioural self regulation [51]. By cognitively redefining the act, individuals may then be able to view intentionally spreading disinformation as permissible, even beneficial, in certain circumstances.

Another reason belief-consistent disinformation may be particularly vulnerable to moral flexibility is if it ‘feels true’. Spreading politically-consistent falsehoods may be judged to be more ethical generally [36], but thinking about how they could be true [5] or might become true [4] can further amplify any partisan effects. Adjustments in moral judgements have been found to predict intention to interact with content on social media [4,7]. Where belief-consistent disinformation is felt to be ‘true’ in some form, people may judge it less harshly than other types of disinformation, even when they are aware it is false or inaccurate.

### Research aims and hypotheses

The present research sought to understand whether people are more lenient towards spreading disinformation when it presents a belief-consistent message and whether such leniency influences their digital interactions with the content (e.g. ‘liking’ or sharing). To demonstrate the role of belief-consistency (rather than partisanship or ideology, for example), consistency with beliefs was evaluated in two studies for two distinct areas–trust in the UK government’s handling of the COVID-19 pandemic, and perceived seriousness of the COVID-19 virus. While beliefs about the COVID-19 virus were influenced by political affiliations in some countries, there was evidence to suggest that that was not the case in the United Kingdom [52] where this data was collected. Therefore, while both pairs of belief types and their corresponding disinformation narratives were related to COVID-19, the relationships themselves in predicting spread intentions and judgements were expected to differ. In study 1 it was predicted that:

**H1.** Individuals would be more likely to interact with misinformation that was consistent with their beliefs.

**H1a.** Individuals who have lower trust in the UK government’s handling of COVID-19 would report a greater likelihood of interacting with misinformation that is unfavourable towards the government than individuals reporting higher trust in the government.

**H1b.** Individuals who have higher trust in the UK government’s handling of COVID-19 would report a greater likelihood of interacting with misinformation that is favourable towards the government than individuals reporting lower trust in the government.

**H1c.** Individuals who believe COVID-19 to be lower risk would report a greater likelihood of interacting with misinformation that minimises COVID-19 risk than those who believe COVID-19 to be higher risk.

**H1d.** Individuals who believe COVID-19 to be higher risk would report a greater likelihood of interacting with misinformation that maximises COVID-19 risk than those who believe COVID-19 to be lower risk.

H2. After learning content is false or misleading, individuals would judge belief-consistent disinformation as being more morally acceptable to spread.

**H2a.** Individuals reporting lower trust in the UK government’s handling of COVID-19 would judge the sharing of disinformation that is unfavourable towards the government as more morally acceptable than individuals reporting higher trust in the government.

**H2b.** Individuals reporting higher trust in the UK government’s handling of COVID-19 would judge the sharing of disinformation that is favourable towards the government as more morally acceptable than those reporting lower trust in the government.

**H2c.** Individuals who believe COVID-19 to be lower risk would judge the sharing of disinformation that minimises COVID-19 risk as more morally acceptable than those who believe COVID-19 to be higher risk.

**H2d**. Individuals who believe COVID-19 to be higher risk will judge the sharing of disinformation that maximises COVID-19 risk as more morally acceptable than those who believe COVID-19 to be lower risk.

Study 2 extends on this by focusing on the influence of belief-consistency on moral evaluations of misinformation (e.g. where participants were not explicitly told the content was untrue). A scale developed for this study was also used in place of ‘interactions’. This measured users’ potential contribution to the social media spread of misinformation based on their intentions to engage in different types of interactions (e.g. those that amplify such as ‘liking’ as well as those that may help reduce onwards spread such as ‘reporting’). The role of moral judgements as a mediator between beliefs and spread will also be addressed.

## Study 1

### Method

#### Materials & procedure

The study was conducted using the Qualtrics online survey platform, with participants drawn from the Prolific research panel. It was advertised as a study about interactions with COVID-19 related social media content. Ethical approval for both studies in this paper came from the University of Westminster Psychology Ethics Committee (ETH2021-0777). Anonymous participants over the age of 18 were asked to select whether they did or did not consent through an electronic form. Participants who did not consent were not able to proceed with the study. After reading the information sheet and giving consent, basic demographic information was collected, including political affiliation. However, analysis along political lines will not be featured in the present paper.

Participants were then asked to complete two scales intended to capture the aforementioned beliefs relating to COVID-19. This included an adapted version of the Citizen Trust in Government Organisation scale [53]. This was tailored to measure trust in the UK government’s handling of the COVID-19 pandemic (e.g. ‘When it concerns the handling of the COVID-19 pandemic in the UK, the government are capable’). Level of agreement with nine statements were measured using a 7-point scale (from ‘Strongly disagree’ to ‘Strongly agree’). Overall score on the Citizen Trust in Government Organisation scale was computed as the mean of all 9 items. The final scale had acceptable reliability (*M =* 3.4; *SD* = 1.6; α = .97). Participants also completed the COVID-19 Perceived Risk Scale [54]. Responses were given on a 5-point scale (from ‘Negligible’ to ‘Very high’). Overall score on the COVID-19 Perceived Risk Scale was computed as the mean of all 8 items. This scale also had acceptable reliability (*M =* 3.02, *SD =* 0.66, α = .83).

Participants were presented with a series of 12 social media posts (including user-generated content and posts containing images). These were sourced from fact checking websites such as Full Fact or were social media content that matched this fact checked content. All items contained either false or misleading information for the time the study took place, for example imagery used out of context or incorrect statistics. Each item was either related to the performance of the UK Government during the pandemic or about COVID-19’s general risk. They had been previously piloted to assign them to opposing categories (the results of the pilot can be seen in the S1 File). Specifically, three images framed the UK Government in an unfavourable light and three were favourable towards the UK government. Another three minimised the risk of COVID-19 and the final three maximised the risk using inaccurate or misleading information. The stimuli can be seen in the S2 File.

Initially, participants were not informed that the stimuli were false or misleading. Each of the 12 items were presented at random and participants were asked to imagine a friend on Facebook had shared the content. Participants were then asked to rate their likelihood of ‘Liking’, ‘Sharing privately’ (e.g. to a friend or a private Facebook group) or ‘Sharing publicly’ (e.g. to their own public newsfeed). Responses were given on a 7-point scale (from ‘extremely unlikely’ to ‘extremely likely’). Scores for each item were summed to create ‘interaction’ scores. Overall, the ‘interaction’ scores for each of the four stimuli sets had very good reliability (*α* = 0.86 to *α =* 0.93).

Finally, participants were informed that the previously seen content had been flagged as problematic by independent fact checkers for being untrue or taken out of context. All 12 items were presented again, however, participants were instead asked to judge how morally acceptable it was for others to share the post. Responses were given on a 7-point scale (from ‘extremely unacceptable’ to ‘extremely acceptable’). Data, analysis syntax and materials for both studies are available at osf.io/rw8jv.

#### Participants

Prolific was used to recruit participants in England with active Facebook accounts on the 11^th^ January, 2021. Each participant was paid £1.00 for their participation. Only participants from England were used so that all participants were rating the same government’s handling of the pandemic. In their analysis, Duffy et al. found divisions in the UK (in relation to the beliefs measured here) aligned with both party and ‘Leave’ / ‘Remain’ identities [52]. Therefore, to ensure a balance, recruitment quotas were equally distributed across self-reported political ideology and vote in the EU referendum. Data were collected on 14^th^ January, 2021. Initially, 231 participants were recruited however, nine did not progress past demographic questions and so were removed. Two participants were removed for not having Facebook accounts. Two further participants were removed for having no variance in their responses to all interaction and moral judgement questions (as well as trust questions for one participant), indicating inauthentic responses. Demographic information for the final 218 participants is found in Table 1.

**Table 1:** Participant demographics.

	*N*	*%*
Total	218	100
Gender		
Female	132	60.6
Male	85	39.0
Non-Binary	1	0.5
Education completed		
Less than GCSEs	2	0.9
GCSEs	30	13.8
A-Levels	42	19.3
Bachelor’s Degree	102	46.8
Master’s Degree	38	17.4
Doctoral Degree	3	1.4
Other	1	0.5
Political Party		
Conservatives	87	39.9
Labour	93	42.7
Liberal Democrats	5	2.3
Other	16	7.3
Unsure	17	7.8

### Results

Descriptive statistics are included in Table 2.

**Table 2:** Summary of descriptive statistics.

					Range			
	N	M	SD	α	Potential	Actual	Skewness	Kurtosis
Age	218	40.98	14.31			19–81	0.48	-0.76
Trust in Government	218	3.40	1.60	.97	1.00–7.00	1.00–6.89	0.10	-1.21
COVID-19 Perceived Risk	217	3.02	0.66	.83	1.00–5.00	1.13–4.63	-0.23	-0.30
Favourable								
Interaction	654	2.09	1.51		1.00–7.00	1.00–7.00	1.40	1.02
Moral Acceptability	654	3.48	1.85		1.00–7.00	1.00–7.00	0.26	-1.01
Unfavourable								
Interaction	653	2.28	1.69		1.00–7.00	1.00–7.00	1.27	0.62
Moral Acceptability	654	3.02	1.78		1.00–7.00	1.00–7.00	0.67	-0.62
Minimising								
Interaction	654	1.82	1.44		1.00–7.00	1.00–7.00	1.86	2.58
Moral Acceptability	654	2.44	1.70		1.00–7.00	1.00–7.00	1.10	0.20
Maximising								
Interaction	218	2.08	1.52		1.00–7.00	1.00–7.00	1.42	1.11
Moral Acceptability	218	3.00	1.81		1.00–7.00	1.00–7.00	0.58	-0.76

To reflect the hierarchical structure of the data, misinformation items (level-1) were nested within participants (level-2). All continuous variables were mean centred. For each set of analysis, six models were built on R using the lme4 package. After the unconditional model (Model 0), control variables of age (mean centred) and gender (dummy variable) were added as fixed effects (Model 1). As level-2 variables of interest, mean centred ‘Trust’ and ‘Risk’ variables were added as fixed effects (Model 2). At level-1, a misinformation category variable was added as a fixed effect (coded as a repeated measure), grouping the stimuli by themes and stances (Model 3A). Next, this category variable was allowed to be random (Model 3B). Finally, cross-level interaction effects were run between the belief (Trust and Risk) variables and misinformation category themes (Model 4), followed up by simple slopes (full tables of which can be found in the S3 File). To test the first group of hypotheses, likelihood of interacting with misinformation was entered as the Dependent Variable (DV). As shown in model 2 (Table 3), beliefs (e.g. ‘Trust’ and ‘Risk’) did not predict interactions with misinformation generally. However, after misinformation categories are introduced (model 3a), model 4 tests for the presence of significant cross-level interaction effects between beliefs and misinformation categories (3 themes entered as dummy variables). Where present, this would suggest that the misinformation categories moderate the relationship between the specific belief and intentions to interact.

**Table 3:** Multilevel model parameters for likelihood of interacting with misinformation.

	Model 2 (*N* = 216)		Model 3B (*N* = 216)		Model 4 (*N* = 216)	
	Est (*SE*)	95% C.I.	Est (*SE*)	95% C.I.	Est (*SE*)	95% C.I.
**Level 1**						
Intercept	0.19 (0.11)	[-0.03, 0.40]	0.19 (0.12)	[-0.04, 0.42]	0.19 (0.12)	[-0.04, 0.42]
MT: Favourable			0.01 (0.07)	[-0.12, 0.14]	0.01 (0.06)	[-0.11, 0.13]
MT: Unfavourable			0.20* (0.09)	[0.02, 0.38]	0.20* (0.08)	[0.04, 0.36]
MT: Minimising			-0.27**(0.09)	[-0.44, -0.10]	-0.27** (0.09)	[-0.44, -0.10]
**Level 2**						
Gender (Female–Male)	-0.31* (0.14)	[-0.59, -0.02]	-0.28* (0.14)	[-0.56, 0.00]	-0.28* (0.14)	[-0.56, -0.00]
Age	0.01 (0.01)	[-0.01, 0.01]	0.01 (0.01)	[-0.01, 0.01]	0.01 (0.01)	[-0.01, 0.01]
Trust	0.01 (0.04)	[-0.08, 0.10]	0.01 (0.04)	[-0.08, 0.10]	0.06 (0.05)	[-0.04, 0.16]
Risk	0.21 (0.11)	[0.00, 0.42]	0.21 (0.11)	[0.00, 0.42]	0.38 (0.12)	[0.14, 0.62]
**Cross-level interaction**						
Trust * MT: Favourable					0.25*** (0.04)	[0.17, 0.33]
Trust * MT: Unfavourable					-0.41*** (0.05)	[-0.51, -0.31]
Trust * MT: Minimising					-0.02 (0.05)	[-0.12, 0.09]
Risk * MT: Favourable					-0.14 (0.09)	[-0.33, 0.04]
Risk * MT: Unfavourable					0.09 (0.12)	[-0.33, 0.15]
Risk * MT: Minimising					-0.48*** (0.13)	[-0.74, -0.23]
**Variance components**						
Residual variance	1.50 (SD = 1.22)		0.96 (SD = 0.98)		0.96 (SD = 0.98)	
Intercept variance	0.90 (SD = 0.95)		1.04 (SD = 1.02)		1.02 (SD = 1.01)	
MT: Favourable variance			0.33 (SD = 0.58)		0.17 (SD = 0.41)	
MT: Unfavourable variance			1.17 (SD = 1.08)		0.76 (SD = 0.87)	
MT: Minimising variance			1.04 (SD = 1.02)		0.95 (SD = 0.98)	
**Additional Information**						
REML criterion (number of parameters)	8869.30 (7)		8403.28 (19)		8310.20 (25)	
AIC; BIC	8883.26; 8924.28		8441.28; 8552.62		8360.71; 8506.70	
ICC	0.38		0.59		0.57	
*df*	2584		2572		2566	
Marginal R^2^; Conditional R^2^	0.02; 0.39		0.03; 0.61		0.09; 0.61	

*Note*. MT = ‘Misinformation Theme’, entered as dummy variables (where 0 = ‘Maximising’ misinformation type).

*p < .05

** *p* < .01

*** *p* < .001.

The first part of H1 outlined the relationship between level of trust in the government’s handling of COVID-19 and intentions to spread government related misinformation (either ‘favourable’ or ‘unfavourable’). In model 4, cross-level interaction effects were present between ‘trust’, and both ‘favourable’ (*B* = 0.25, 95% CI [0.17, 0.33]) and ‘unfavourable’ misinformation categories (*B* = -0.41, 95% CI [-0.51, -0.31]). This suggests that for both types of government-related misinformation, any relationship with ‘trust’ is not only significantly stronger than for ‘maximising’ misinformation (where *B* = 0.06, 95% CI [-0.04, 0.16]), these relationships occur in opposite directions. Analysis of simple slopes confirmed that participants with lower ‘trust’ in the government’s handling of the pandemic were more likely to interact with misinformation framing the government ‘unfavourably’ than those who had higher ‘trust’, (*B* = -0.29, 95% CI [-0.41, -0.18]). Moreover, higher levels of ‘trust’ predicted increased likelihood of interacting with misinformation that appeared ‘favourable’ towards the government compared to lower ‘trust’, (*B* = 0.20, 95% CI [0.09, 0.31]). The findings suggest that these opposing beliefs about the government may predict interactions with two distinct types of misinformation (pro- and anti- government), supporting both H1a and H1b.

The other relationships defined in H1 focused on misinformation relating to the threat of the COVID-19 virus and beliefs about perceived COVID-19 risk. In model 4, the coefficient for ‘risk’ was significant (*B* = 0.38, 95% CI [0.14, 0.62]), suggesting that higher levels of perceived risk were related to increased likelihood of interacting with ‘maximising’ misinformation. Furthermore, a cross-level interaction effect was found between ‘risk’ and the ‘minimising’ misinformation category (*B* = -0.48, 95% CI [-0.74, -0.23]). However, analysis of simple effects showed that lower levels of perceived risk were not, however, related to increased likelihood of interacting with ‘minimising’ misinformation (*B* = -0.16, 95% CI [-0.45, 0.13]), which may in part be due a very low level of intention to interact with the content overall. Nonetheless, H1c is rejected while H1d is accepted. Content containing false or misleading information when presenting COVID-19 as high risk was more likely to be interacted with by users whose beliefs were consistent with this risk evaluation. To test H2, moral judgements of spreading the content after participants were informed it was untrue was entered as the DV. Again, ‘trust’ and ‘risk’ were not significant predictors in the model (Table 4). However, as before, in model there were some cross-level interaction effects between the beliefs and disinformation categories.

**Table 4:** Multilevel model parameters for moral acceptability of spreading disinformation.

	Model 2 (*N =* 216)		Model 3B (*N =* 216)		Model 4 (*N =* 216)	
	Est (*SE*)	95% C.I.	Est (*SE*)	95% C.I.	Est (*SE*)	95% C.I.
**Level 1**						
Intercept	0.23 (0.13)	[-0.03, 0.49]	0.24 (0.14)	[-0.03, 0.52]	0.24 (0.14)	[-0.03, 0.51]
MT: Favourable			0.49*** (0.09)	[0.32, 0.65]	0.49*** (0.08)	[0.33, 0.65]
MT: Unfavourable			0.03 (0.09)	[-0.15, 0.21]	0.03 (0.08)	[-0.13, 0.19]
MT: Minimising			-0.56***(0.09)	[-0.74, -0.37]	-0.56***(0.09)	[-0.73, -0.38]
**Level 2**						
Gender (Female–Male)	-0.38* (0.17)	[-0.71, -0.04]	-0.38* (0.17)	[-0.71, -0.05]	-0.38* (0.17)	[-0.71, -0.05]
Age	-0.01 (0.01)	[-0.02, 0.00]	-0.01 (0.01)	[-0.02, 0.00]	-0.01 (0.01)	[-0.02, 0.00]
Trust	-0.02 (0.05)	[-0.13, 0.08]	-0.09 (0.05)	[-0.19, 0.02]	-0.01 (0.06)	[-0.12, 0.11]
Risk	-0.24 (0.13)	[-0.49, 0.01]	-0.27* (0.13)	[-0.51, -0.02]	-0.02 (0.14)	[-0.30, 0.26]
**Cross-level interaction**						
Trust * MT: Favourable					0.25*** (0.05)	[0.15, 0.35]
Trust * MT: Unfavourable					-0.37*** (0.05)	[-0.47, -0.27]
Trust * MT: Minimising					0.05 (0.06)	[-0.06, 0.16]
Risk * MT: Favourable					-0.06 (0.12)	[-0.30, 0.18]
Risk * MT: Unfavourable					-0.20 (0.14)	[-0.44, 0.04]
Risk * MT: Minimising					-0.63*** (0.14)	[-0.89, -0.36]
**Variance components**						
Residual variance	1.99 (SD = 1.41)		1.31 (SD = 1.15)		1.31 (SD = 1.15)	
Intercept variance	1.30 (SD = 1.14)		1.46 (SD = 1.21)		1.42 (SD = 1.19)	
MT: Favourable variance			0.70 (SD = 0.84)		0.55 (SD = 0.74)	
MT: Unfavourable variance			0.90 (SD = 0.95)		0.56 (SD = 0.75)	
MT: Minimising variance			1.01 (SD = 1.15)		0.82 (SD = 0.92)	
**Additional Information**						
REML criterion (number of parameters)	9619.00 (7)		9166.10 (19)		9080.00 (25)	
AIC; BIC	9633.00; 9674.02		9204.11; 9315.46		9129.27; 9276.48	
ICC	0.40		0.59		0.56	
*df*	2585		2573		2567	
Marginal R^2^; Conditional R^2^	0.03; 0.41		0.07; 0.62		0.11; 0.61	

*Note*. MT = ‘Misinformation Theme’, entered as dummy variables (where 0 = ‘Maximising’ misinformation type).

*p < .05

** *p* < .01

*** *p* < .001.

There were again significant cross-level interaction effects between trust-related beliefs in the context of favourable (*B* = 0.25, 95% CI [0.15, 0.35]) and unfavourable disinformation (*B* = -0.37, 95% CI [-0.47, -0.27]). Analysis of simple effects showed that those with higher trust in the government’s handling of the pandemic judged spreading ‘favourable’ disinformation as more morally acceptable compared to those with lower levels of trust (*B* = 0.16, 95% CI [0.03, 0.30]). Those with lower trust instead were more likely to judge ‘unfavourable’ disinformation about the government as more morally acceptable compared to those with high trust (*B* = -0.30, 95% CI [-0.42, -0.17]). Again, both H2a and H2b are accepted, with disinformation consistent with beliefs being viewed as more acceptable to spread, even when known to be untrue. However, as the coefficient for ‘risk’ was not significant, this time there was no evidence of an effect of belief-consistency for moral judgements of ‘maximising’ disinformation (*B* = -0.02, 95% CI [-0.30, 0.26]). However, model 4 showed a cross-level interaction effect between ‘minimising’ disinformation and risk-related beliefs (*B* = -0.63, 95% CI [-0.89, -0.36]). Those who perceived COVID-19 to be lower-risk were more morally accepting than others of spreading disinformation that supported this view (*B* = -0.71, 95% CI [-1.03, -0.39]). Therefore, only H2c is accepted, providing some further support that belief-consistent disinformation may be viewed as more acceptable to spread.

### Discussion

This study has provided some support for H1. Firstly, belief-consistency appeared to play a role in intentions to interact with misinformation for three out of four categories. In particular, the direction of the relationship between trust in the UK government’s handling of the COVID-19 pandemic and interaction with misinformation about the same issue was dependent on the sentiment expressed within the content itself. This suggests suggesting that the level of closeness with viewers beliefs plays a role in its spread. Belief-consistency also predicted intentions to interact with misinformation that expressed the threat of COVID-19 as serious (but using misleading information to do so), but perceived risk did not have a significant relationship with intentions to interaction with misinformation that minimised the risk of COVID-19. Overall, the findings lend support to belief-consistency playing a role in misinformation interactions.

Belief-consistency was also associated with moral judgements of disinformation in three of the four hypothesised relationships (H2). For both types of government related disinformation and for disinformation that minimised the risk of COVID-19, this suggests that participants made moral judgements regarding other people spreading disinformation based on how closely the content itself matched their beliefs.

## Study 2

Study 1 showed that the belief-consistency of misinformation influenced how likely people were to interact with it. However, where there was low intention to interact overall (e.g. misinformation which minimised the threat of COVID-19), the relationship with a corresponding belief was not significant. It also found that the belief-consistency of content known to be false or misleading influences how morally acceptable people feel it is to share on social media. Study 2 therefore set out to understand whether this flexibility extends to misinformation (e.g. where the content is not necessarily known to be disinformation). It also looked at whether these judgements influenced not just the digital interactions that would increase the spread of content, but also considered attempts to prevent its spread by using a scale designed for this study. To simplify the design of the study, only government related stimuli were presented to participants. Similar to Study 1, it was hypothesised that:

**H1.** Individuals would be more likely to contribute to the spread of misinformation when it was consistent with their beliefs.

**H1a**: Individuals who have lower trust in government handling of COVID-19 will report a greater likelihood of contributing to the spread of misinformation that undermines the government.

**H1b**: Individuals who have higher trust in government handling of COVID-19 will report a greater likelihood of contributing to the spread of misinformation that supports the government.

**H2.** Individuals would be more likely to judge the sharing of belief-consistent misinformation to be morally acceptable.

**H2a:** Individuals with lower trust in the government will report the sharing of misinformation that undermines the government as more morally acceptable than those with higher trust in the government.

**H2b:** Individuals with higher trust in the government will report the sharing of misinformation that supports the government as more morally acceptable than those with lower trust in the government.

**H3.** Moral judgements of belief-consistent misinformation would mediate the relationship between beliefs and the likelihood of spreading belief-consistent misinformation.

**H3a:** Moral judgement of sharing ‘government undermining’ misinformation will mediate the relationship between low trust and increased likelihood of spreading ‘undermining’ misinformation.

**H3b:** Moral judgement of sharing ‘government supporting’ misinformation will mediate the relationship between high trust and increased likelihood of spreading ‘supporting’ misinformation.

Hypotheses for this study were pre-registered and can be seen at https://aspredicted.org/3KP_1KC. Hypotheses 1 and 2 were tested using multi-level models, while hypothesis 3 was tested using a multi-level mediation model. This was a departure from the pre-registered analysis on the recommendation of a reviewer. The original pre-registered analysis was also conducted, and can be seen in the S4 File, however the outcomes of both analyses were the same.

### Method

#### Materials & procedure

The procedure and materials for this study were replicated from study one, with any changes noted. Six of the stimuli from the original study were presented at random to participants. These were false or misleading items that were either ‘Favourable’ or ‘Unfavourable’ towards the UK Government. As before, participants were not explicitly informed that the content was false or misleading. They were asked how likely they would be to engage with a series of eight actions if the image appeared on their social media feed. These actions formed a ‘Social Media Spread’ scale (Table 5), incorporating actions which contribute to (e.g. ‘repost the content on a personal social media account’) or may help to reduce (e.g. ‘report the message to the platform’) the overall reach of content on social media. Responses were taken using an 11-point scale from ‘not at all likely’ to ‘extremely likely’ and items relating to reduced spread of content were reverse scored.

**Table 5:** Items for social media spread scale.

	Items
PREFIX: If this image came up on your social media feed, how likely is it you would engage with the following actions?	
S1	Like or upvote the content
S2	Comment in agreement / support
S3	Repost the content on a personal social media account (e.g. “retweet”)
S4	Send the content directly to one other person
S5	Share the content with a group of other people (e.g. WhatsApp group)
S6 (R)	Report the message to the platform
S7 (R)	Post comment asking for content to be taken down
S8 (R)	Directly contact the poster to ask them to remove

(R) reverse score.

When participants were asked to rate how morally acceptable they felt sharing the content was, this time they were not informed that the items were misleading until the debrief. Responses were given on an 11-point scale from ‘not at all acceptable’ to ‘completely morally acceptable’.

#### Participants

An initial sample of 302 participants, all social media users based in England, were recruited through Prolific on the 28^th^ October, 2021. Each was paid £1.00 for their participation. Eighteen participants were automatically removed from the study due to using an incompatible device. In the data cleaning stage, one participant was removed for not consenting, another for not meeting the recruitment criteria regarding current location, while a further three did not complete the study. Qualtrics’ proprietary software flagged four participants as suspicious and so they were removed. A further 24 participants were also removed for a lack of variance in their responses on the ‘Citizen Trust’ and ‘Perceived Risk’ scales that suggested inauthentic responses. The demographics for the remaining 251 participants are found in Table 6.

**Table 6:** Participant demographics.

	N	%
Total	251	100
Gender		
Female	163	64.9
Male	83	33.1
Non-Binary	3	1.2
Prefer not to say	2	0.8
Education completed		
Less than GCSEs	4	1.6
GCSEs	25	10.0
A-Levels	78	31.1
Bachelor’s Degree	96	38.2
Master’s Degree	42	16.7
Doctoral Degree	2	0.8
Other	4	1.6
Political Party		
Conservatives	90	35.9
Labour	85	33.9
Liberal Democrats	6	2.4
Other	34	13.5
Unsure / Would not vote	32	12.7
Prefer not to say	4	1.6

### Results

Descriptive statistics are included in Table 7. All scales had acceptable reliability.

**Table 7:** Summary of descriptive statistics.

					Range			
	N	M	SD	α	Potential	Actual	Skewness	Kurtosis
Age	251	35.47	13.29			18–71	0.61	-0.70
Trust in Government	251	3.37	1.46	.96	1–7	1.11–6.67	0.25	-0.96
COVID-19 Perceived Risk	251	2.76	0.69	.84	1–5	1.13–4.63	-0.06	-0.21
Spread Likelihood								
Favourable	753	5.50	1.19		1–11	2.50–11	1.80	3.65
Unfavourable	753	5.80	1.66		1–11	1.62–11	1.28	1.59
Moral Judgement								
Favourable	753	6.93	2.98		1–11	1–11	-0.34	-0.71
Unfavourable	753	5.45	3.27		1–11	1–11	0.21	-1.07

To test whether the belief-consistency of misinformation increased the likelihood that social media users would contribute to its spread (H1), the multilevel model steps from study 1 were again applied. Model 2 (Table 8) again indicates that beliefs (e.g. ‘trust’ and ‘risk’) did not predict intentions to spread misinformation generally. However, in the final model (model 4), ‘Trust’ was a significant predictor of spread (*B* = -0.30, 95% CI [-0.43, -0.25]), suggesting that lower ‘trust’ in government was related to increased likelihood of spreading ‘unfavourable’ misinformation. A cross-level interaction effect suggests the misinformation category variable moderated the relationship between ‘trust’ and spread (*B* = 0.49, 95% CI [0.60, 0.37]). Analysis of simple slopes suggest that higher trust predicted greater likelihood than others of spreading ‘favourable’ misinformation about the government (*B* = 0.18, 95% CI [0.09, 0.27]). Levels of belief-consistency with misinformation may therefore influence how people contribute to its wider spread. Both H1a and H1b are supported.

**Table 8:** Multilevel model parameters for likelihood of spreading misinformation.

	Model 2 (*N* = 246)		Model 3B (*N* = 246)		Model 4 (*N =* 246)	
	Est (*SE*)	95% C.I.	Est (*SE*)	95% C.I.	Est (*SE*)	95% C.I.
**Level 1**						
Intercept	-0.05 (0.11)	[-0.27, 0.17]	0.13 (0.13)	[-0.12, 0.38]	0.14 (0.12)	[-0.10, 0.38]
MT: Favourable			-0.31*** (0.10)	[-0.50, -0.12]	-0.32*** (0.08)	[-0.49, -0.16]
**Level 2**						
Gender (Female–Male)	0.10 (0.14)	[-0.17, 0.37]	0.06 (0.13)	[-0.20, 0.31]	0.06 (0.13)	[-0.20, 0.31]
Age	-0.01* (0.01)	[-0.02, 0.00]	-0.01* (0.01)	[-0.02, 0.00]	-0.01* (0.01)	[-0.02, 0.00]
Trust	-0.06 (0.05)	[-0.16, 0.03]	0.06 (0.04)	[-0.03, 0.15]	-0.30*** (0.06)	[-0.43, -0.18]
Risk	0.15 (0.09)	[-0.03, 0.34]	0.07 (0.09)	[-0.11, 0.24]	0.35** (0.13)	[0.11, 0.60]
**Cross-level interaction**						
Trust * MT: Favourable					0.49*** (0.06)	[0.37, 0.60]
Risk * MT: Favourable					-0.39** (0.12)	[-0.62, -0.15]
**Variance components**						
Residual variance	1.30 (SD = 1.14)		0.75 (SD = 0.86)		0.75 (SD = 0.86)	
Intercept variance	0.78 (SD = 0.88)		1.91 (SD = 1.38)		1.59 (SD = 1.26)	
*MT*: *Favourable* variance			1.77 (SD = 1.33)		1.20 (SD = 1.10)	
**Additional Information**						
REML criterion (number of parameters)	4972.40 (7)		4634.50 (10)		4567.40 (12)	
AIC; BIC	4986.40; 5023.48		4654.61; 4707.58		4591.39; 4654.96	
ICC	0.38		0.64		0.61	
*df*	1469		1466		1464	
Marginal R^2^; Conditional R^2^	0.03; 0.39		0.02; 0.65		0.11; 0.65	

*Note*. MT = ‘Misinformation Theme’, entered as dummy variables (where 0 = ‘Unfavourable’ misinformation type).

*p < .05

** *p* < .01

*** *p* < .001.

Next, moral judgements of spreading misinformation, prior to learning that the content is false or misleading, was entered as the DV. As shown in Table 9, while beliefs were again not significant predictors of moral judgements of misinformation generally (model 2), ‘trust’ was a significant predictor of moral judgements in the final model (*B* = -0.74, 95% CI [-0.43, -0.25]). This suggests that ‘unfavourable’ misinformation was judged to be more morally acceptable to spread by those with low trust compared to others. A cross-level interaction effect suggests the misinformation category variable moderated the relationship between ‘trust’ and moral judgements (*B* = 1.20, 95% CI [0.96, 1.45]). Analysis of simple slopes suggest that spreading favourable misinformation was viewed as more morally acceptable by those with high trust compared to those with low trust (*B* = 0.47, 95% CI [0.23, 0.70]). Thus, H2a and H2b are supported.

**Table 9:** Multilevel model parameters for moral acceptability of spreading misinformation.

	Model 2		Model 3B		Model 4	
	Est (*SE*)	95% C.I.	Est (*SE*)	95% C.I.	Est (*SE*)	95% C.I.
**Level 1**						
Intercept	0.37 (0.24)	[-0.10, 0.85]	-0.38 (0.26)	[-0.90, 0.14]	-0.36 (0.26)	[-0.87, 0.14]
MT: Favourable			1.52*** (0.21)	[1.10, 1.94]	1.49*** (0.18)	[1.13, 1.84]
**Level 2**						
Gender (Female–Male)	-0.50 (0.30)	[-1.08, 0.09]	-0.51 (0.30)	[-1.09, 0.08]	-0.51 (0.30)	[-1.09, 0.08]
Age	-0.04** (0.01)	[-0.06, -0.01]	-0.04** (0.01)	[-0.06, -0.01]	-0.04** (0.01)	[-0.06, -0.01]
Trust	-0.13 (0.10)	[-0.34, 0.07]	-0.12 (0.10)	[-0.32, 0.08]	-0.74*** (0.12)	[-0.97, -0.50]
Risk	-0.07 (0.20)	[-0.47, 0.33]	-0.08 (0.20)	[-0.48, 0.31]	0.29 (0.24)	[-0.19, 0.77]
**Cross-level interaction**						
Trust * MT: Favourable					1.20*** (0.12)	[0.96, 1.45]
Risk * MT: Favourable					-0.72** (0.26)	[-1.24, -0.21]
**Variance components**						
Residual variance	6.35 (SD = 2.52)		2.84 (SD = 1.68)		2.84 (SD = 1.68)	
Intercept variance	3.64 (SD = 1.91)		6.72 (SD = 2.59)		5.85 (SD = 2.42)	
*MT*: Favourable variance			9.43 (SD = 3.07)		6.10 (SD = 2.47)	
**Additional Information**						
REML criterion (number of parameters)	7292.40 (7)		6742.10 (10)		6657.80 (12)	
AIC; BIC	7306.37; 7343.45		6762.06; 6815.03		6681.77; 6745.34	
ICC	0.37		0.70		0.67	
*Df*	1469		1466		1464	
Marginal R^2^; Conditional R^2^	0.04; 0.39		0.09; 0.73		0.17; 0.73	

*Note*. MT = ‘Misinformation Theme’, entered as dummy variables (where 0 = ‘Unfavourable’ misinformation type).

*p < .05

** *p* < .01

*** *p* < .001.

**Fig 1:**
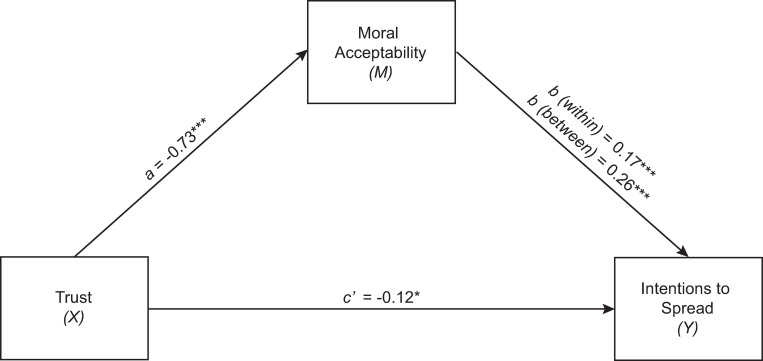
Unstandardised coefficients for the relationship between ‘trust’ and likelihood of spreading ‘unfavourable’ misinformation, mediated by moral judgements of ‘unfavourable’ misinformation. Controlled for ‘risk’ and age. *p < .05. ** p < .01. *** p < .001.

Finally, two multi-level mediation analyses were carried out using the SPSS MLMed macro to test whether moral judgements of spreading the misinformation (which they did not know at that stage was untrue) mediated the relationship between belief-consistency and spread (H3). Full summary tables for both models can be found in the S3 File. The first model predicted likelihood of spreading ‘unfavourable’ misinformation while controlling for age and perceived risk of COVID-19 (both significant coefficients in the previous models). As seen in Fig 1, those with low trust in the Government’s handling of the pandemic were more morally accepting of spreading belief-consistent (e.g. ‘Unfavourable’) misinformation (*a* = -0.73, 95% CI [-0.97, -0.49]). and these moral judgements were subsequently related to a higher likelihood of spread (within effect *b* = 0.18, 95% CI [0.13, 0.21]; between-effect *b* = 0.26, 95% CI [0.21, 0.32]). Based on 5000 bootstrapped samples, the between-indirect effect (*ab* = -0.19) was significantly different from zero (95% *CI* = [-0.27, -0.12]), and partially mediated the between-direct effect between belief consistency (e.g. ‘trust’ level) and spread (*c’* = -0.12, (95% *CI* = [-0.23, -0.00]).

**Fig 2:**
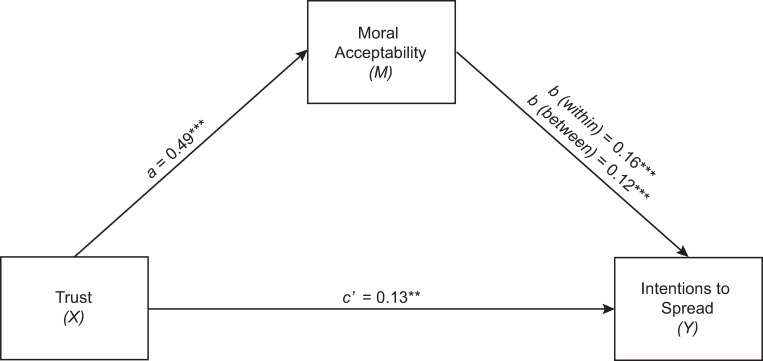
Unstandardised coefficients for the relationship between ‘trust’ and likelihood of spreading ‘favourable’ misinformation mediated by moral judgements of ‘favourable’ misinformation. Controlled for gender (Dummy variable ^a^) and age. *p < .05. ** p < .01. *** p < .001. ^a^ Male = 0, Female = 1. Participant *N* = 246 as 5 participants identifying as non-binary or not disclosing gender were excluded from this analysis.

The second model predicted likelihood of spreading ‘favourable’ misinformation, controlling for age and gender (Fig 2). High trust was related to increased moral acceptance of spreading belief-consistent (e.g. ‘favourable’) misinformation (*a* = 0.49, (95% *CI* = [0.25, 0.73]). This judgement was then related to an increased likelihood of spreading ‘Favourable’ misinformation (within effect *b* = 0.16, 95% CI [0.11, 0.20]; between-effect *b* = 0.12, 95% CI [0.07, 0.16]). Again, there was an indirect effect (*ab* = 0.06) which was significantly different from zero based on 5000 bootstrapped samples (95% *CI* = [0.02, 0.10]). This again partially mediated the relationship between consistent beliefs and contribution to misinformation spread (*c’* = 0.13, 95% CI [0.04, 0.22]).

## General discussion

Two studies were carried out to assess how belief-consistency levels of disinformation may influence spread and related moral judgements. The findings indicate that social media users are more likely than other people to contribute to the spread of misinformation when the message is consistent with their beliefs. Additionally, levels of belief-consistency may also influence users’ moral evaluations of misinformation and disinformation. When misinformation was consistent with beliefs, participants judged it as more acceptable to spread than others, even when participants knew the content was false or misleading (Study 1), and these moral judgements also partially mediated the role between belief-consistency and spread (Study 2).

The findings suggest that people’s beliefs about a specific issue may influence whether they go on to spread belief-consistent misinformation on social media, as well as their moral evaluations of the content. Previous work found people are more likely to believe [19], spread [18], and make positive moral evaluations [6] of false information when it is consistent with their ideology or partisanship. The present research expands on this, by considering that issue-specific political beliefs may not always map cleanly onto partisanship or ideological categories. Indeed, others have shown that interpreting interactions via ideological categories may hide other important factors [21]. The approach used here may provide useful for future misinformation studies looking to disentangle beliefs from ideology.

Furthermore, while previous research has found one-way relationships between susceptibility and issue-related beliefs [28], the present findings indicate that this may be influenced by the presentation of specific stances of misinformation. Here, it was found that for all but one misinformation theme, level of belief-consistency appeared to play a role in users’ likelihood of contributing to its spread. In particular, while Roozenbeek et al. [28] found that higher levels of trust in politicians’ COVID-19 approach was related to increased susceptibility to COVID-19 misinformation, here that was not the case generally. Indeed, as with previous research which found the influence of political attitudes on interactions with related misinformation was dependent on stance [25,26], the relationship between beliefs and intentions to spread misinformation did not appear to be one-way. Indeed, it was found that the stance of misinformation may moderate the relationship between issue-specific beliefs and intentions to spread issue-relevant misinformation. In some instances, this moderation effect entirely switched the direction of the relationship with beliefs. This finding, in hand with previous work [25,26], highlight that the closeness of beliefs to presented misinformation stances are an important consideration when interpreting findings in this area.

Similarly, the work presented here indicates that the narratives presented within misinformation may also offer important context. Here, it was found that specific beliefs relating to COVID-19 may only help explain judgements about relevant narratives of COVID-19 misinformation. Indeed, it was only when the individual misinformation narratives were isolated that the influence of the two COVID-19 related beliefs became clear. This adds to recent work which found narrative-level nuances in how attitudes [23] and political orientation [22] influence misinformation susceptibility. While the term ‘misinformation’ has often been used in a generalised way to reflect a collection of false and misleading information, these findings help demonstrate the potential value of accounting for narrative types. For instance, where prior work has found cross-cultural differences in how issue-related beliefs influence susceptibility to ‘misinformation’ [28], accounting for misinformation narratives may help provide important context. Arguably, it may be important to distinguish between potential factors of misinformation susceptibility and relevance-based factors of susceptibility. Although the present work focuses on spread-related judgements, accounting for belief-consistency effects could also be beneficial to identify true indicators of misinformation susceptibility.

Another important finding was that moral evaluations of misinformation were also influenced by belief-consistency, and in turn influenced intentions to spread it further. This supports a number of recent studies where moral evaluations have been found to play a role in intentions to interact with misinformation [4,7]. Research has also found that thinking about how disinformation could be [5] or might become true [4] may alleviate any moral condemnation of spreading disinformation further, as well as amplifying prior biases. As previous work suggests that belief-consistent misinformation may be perceived as more accurate [34] (perhaps due to said beliefs reflecting what they believe to be ‘true’ [32,33]), participants may have felt that the content was more likely to be true. However, this would not entirely explain the present findings as, at least for moral judgements, the actual veracity of the content did not appear to always matter. Specifically, the relationship between degree of belief-consistency and moral acceptability occurred even when participants were aware the information was misleading. While research suggests that social media users are less likely to spread misinformation if they are prompted to consider accuracy [37], from a motivated reasoning perspective at least, people can achieve accuracy-related goals by other means than identifying veracity [42]. It may be that belief-consistent disinformation may provide other types of accuracy cues that allows it to be judged differently from disinformation generally. Overall, these findings suggest that while people may demonstrate concern about ‘disinformation’, they may be less concerned with disinformation that supports their perceived reality in some way (and in turn, may be more likely to spread it).

One intriguing finding was that belief-consistency of COVID-19 ‘maximising’ disinformation was not a significant predictor of moral judgements but did predict intentions to interact prior to learning the content was false or misleading. As misinformation may be used to express moral emotions [15] and beliefs [55], certain moral concerns may outweigh those relating to veracity. Furthermore, prior research indicates that when people feel false information may benefit someone else [27] or it supports their moral convictions [6] they may be more morally lenient than towards other deceptions. Given that data for study 1 was collected when COVID-19 restrictions were still in place in the UK, making evaluations of ‘maximising’ disinformation may have presented a moral dilemma for some participants. Arguably, the perceived moral implications of spreading disinformation may have been equal to or outweighed by other moral concerns, such as the need to conform to the rules to keep others’ safe. This type of situation could pose a challenge for current efforts to reduce misinformation spread. Research suggests that deliberative reasoning may help users identify misinformation in an unbiased way [40,56] and improve the quality of content users share online [57]. However, there is also evidence to suggest deliberative reasoning can amplify moral hypocrisy [58] and moral judgements favouring ‘the greater good’ [59]. More work is certainly needed to understand how moral dilemmas might influence judgements of disinformation.

What the present findings do indicate is that users may not always feel that the act of spreading belief-consistent disinformation is equal to spreading other types of disinformation. In some instances, this difference may allow users who encounter belief-consistent disinformation to rationalise contributing to its spread. However, as the present findings indicate, individual-level moral leniency may not necessarily lead to users’ actively spreading misinformation. People are motivated to be seen by others as ‘moral’ and therefore may adjust their behaviour accordingly [8]. Although levels of perceived risk of COVID-19 related to how morally acceptable participants’ judged spreading known ‘minimising’ disinformation here, the level of intention to interact with this misinformation was lower overall and belief-consistency was not a significant predictor of intentions to interact with the content. If participants were aware these narratives were associated with misinformation, reputational concerns [45] may have played a part in reducing intentions to interact.

There are of course limitations with the present study. Namely, the present research supposes social media content may induce a sense of ‘right’ or ‘wrong’ that may determine user’s next steps–the ‘affective flashes’ proposed by Haidt [46]. Yet, here participants were asked to make judgements of the disinformation content which may engage moral reasoning processes (potentially involving more deliberative, considered thought). It is, however, supposed that moral intuition has great influence over reasoning outcomes [60] and therefore it may the case that participant responses were reflective of moral intuition. Furthermore, as perceived accuracy was not measured, it cannot be known the extent to which participant responses were influenced by belief or not.

The present work has important implications for developing our understanding of why people spread disinformation on social media. If people make different moral evaluations regarding false or misleading content that supports their beliefs, then it could increase the chance that it is seen as ‘different’ to disinformation generally. If that is the case, even if users care about the accuracy of information online generally, they may perceive belief-consistent disinformation (that feels subjectively accurate) to be ‘an exception’. Given that disseminators of disinformation have targeted users based on personal preferences [13] and social media algorithms ensure users are presented with personally relevant content [16], the potential implications of this are concerning. Specifically, when users encounter disinformation or misinformation within their own feeds, it is likely to be the types of content they are more likely to make exceptions for and help spread.

## Conclusion

While the issue of disinformation spread is not unique to social media platforms, their very nature and design create an environment where disinformation may be spread with relative ease. Understanding the factors influencing users’ own contributions to the onward spread of disinformation (either intentionally or not) is therefore of importance. Here, it was found that the degree to which misinformation was consistent with participants’ beliefs influenced how likely they would be to go on and spread it compared to others. The findings also suggest that belief-consistency was a more important predictor of users contributing to misinformation spread than political affiliation. The present findings also suggest that greater leniency in moral judgements of disinformation may occur in relation to level of belief-consistency. Greater moral acceptance towards spreading belief-consistent misinformation was also found to partially explain intentions to spread the content further. Ultimately, if social media users are more lenient towards belief-consistent disinformation, they may not feel it is harmful or ‘wrong’ to spread (even if they acknowledge disinformation to be a problem generally). Further research is needed to understand the implications of this on intervention work.

## Supporting information

S1 File Pilot study results. (DOCX)

S2 File Stimuli for Studies 1 and 2. (DOCX)

S3 File Supporting information for Studies 1 and 2. (DOCX)

S4 File Study 2 –Pre-registered analyses. (DOCX)
