# Supplementary material for: Moral leniency towards belief-consistent disinformation may help explain its spread on social media
Source: PLoS One. 2023 Mar 22;18(3):e0281777. doi: 10.1371/journal.pone.0281777 (PMC10032519; doi:10.1371/journal.pone.0281777)
Supplement: S1 File — (DOCX) [file pone.0281777.s001.docx]

S1. Pilot study results

The materials used in studies 1 and 2 were piloted to ensure the correct allocation of stimuli (e.g. ‘favourable’ or ‘unfavourable’) and select the most representative items for each set. It was also intended that the results would confirm that paired stimuli sets were significantly different from one another.

Materials

A selection of 16 items that were either related to the performance of the UK government or regarding the risk of COVID-19 were piloted. Within each grouping, the items were divided into opposing stances (e.g. four items that were favourable towards the government, four items that undermined the government). All items contained either false or misleading information for the time the study took place, for example imagery used out of context or incorrect statistics.

Participants

23 participants (6 males) aged 20-65 (*M =* 28.91*, SD =* 12.32) were recruited for the study via social media. Participants were required to be current residents of England.

Procedure

The study was hosted online using the survey platform Qualtrics. After basic demographic questions, participants rated how favourable the images relating to the UK government were on an 11-point scale (1-‘Very unfavourable’ to 11-‘Very favourable’). They then rated the COVID-19 images in terms of the level of risk illustrated using an 11-point scale from ‘Not at all risky’ to ‘Very high risk’.

Results

Mean favourability scores for government-related images are displayed in Table 1. The three items with the lowest scores were allocated to the ‘unfavourable’ stimuli set, while the three highest scores were allocated to the ‘favourable’ stimuli set.

**S1A Table. Favourability ratings of government-themed stimuli**

| *Item* | *N* | *Minimum* | *Maximum* | *Mean* | *SD* |
| --- | --- | --- | --- | --- | --- |
| FG 1* | 23 | 5 | 10 | 8.39 | 1.34 |
| FG 2* | 23 | 6 | 11 | 8.57 | 1.53 |
| FG 3 | 23 | 1 | 9 | 3.91 | 2.23 |
| FG 4* | 23 | 5 | 11 | 8.57 | 1.62 |
| UG 1* | 23 | 1 | 4 | 2.04 | 1.02 |
| UG 2* | 23 | 1 | 6 | 1.96 | 1.61 |
| UG 3* | 23 | 1 | 6 | 1.96 | 1.3 |
| UG 4 | 23 | 1 | 9 | 3.35 | 2.12 |

Abbreviation, FG – ‘Favourable’, UG – ‘Unfavourable’. * items in final selection.

The mean risk perception scores for the COVID-19 images are presented in Table 2. The three highest rated images and three lowest rated images were selected for ‘minimising’ and ‘maximising’ stimuli sets.

**S1B Table. Risk ratings of COVID-19 related stimuli**

|  | *N* | *Minimum* | *Maximum* | *Mean* | *SD* |
| --- | --- | --- | --- | --- | --- |
| MinCV 1 | 23 | 1 | 8 | 3.43 | 1.88 |
| MinCV 2* | 23 | 1 | 10 | 3.09 | 2.15 |
| MinCV 3* | 23 | 1 | 8 | 3.04 | 2.14 |
| MinCV 4* | 23 | 1 | 7 | 3.22 | 1.7 |
| MaxCV 1* | 23 | 4 | 10 | 7.04 | 1.64 |
| MaxCV 2 | 23 | 1 | 10 | 6.48 | 2.11 |
| MaxCV 3* | 23 | 5 | 11 | 9.35 | 1.58 |
| MaxCV 4* | 23 | 2 | 10 | 7.87 | 1.58 |

Abbreviations, MinCV – ‘Minimising COVID-19’, MaxCV – ‘Maximising COVID-19’. * items in final selection.

An overall mean score was calculated for each stimuli set. Paired *t-*tests were then carried out to confirm that the stimuli sets were significantly different. The ‘unfavourable’ (*M =* 1.99, *SD =* 1.52) and ‘favourable’ (*M =* 8.51, *SD =* 1.33) sets were significantly different, *t*(22) = 13.99, *p* < .001, *d =* 2.91. ‘Minimising CV19’ (*M =* 3.12, *SD =* 1.59) was also assigned a significantly lower risk rating than ‘Maximising CV19’ (*M =* 8.09, *SD =* 1.11), *t*(22) *=* 11.83, *p* < .001, *d =* 2.47. The large effect sizes suggest that both pairs of stimuli sets are distinctly different (e.g. in either favourability or risk).

Discussion

Six images were selected as being the most or least favourable towards the UK Government and size for representing COVID-19 as the highest or lowest risk. The difference between each pair of stimuli sets was significant, suggesting that these items are suitable to represent distinct ‘themes’ of misinformation. The final selection of items can be found in S2.
