# Supplementary material for: Moral leniency towards belief-consistent disinformation may help explain its spread on social media
Source: PLoS One. 2023 Mar 22;18(3):e0281777. doi: 10.1371/journal.pone.0281777 (PMC10032519; doi:10.1371/journal.pone.0281777)

S2. Stimuli for Studies 1 and 2

‘Unfavourable’ towards the UK Government (Study 1 & 2)


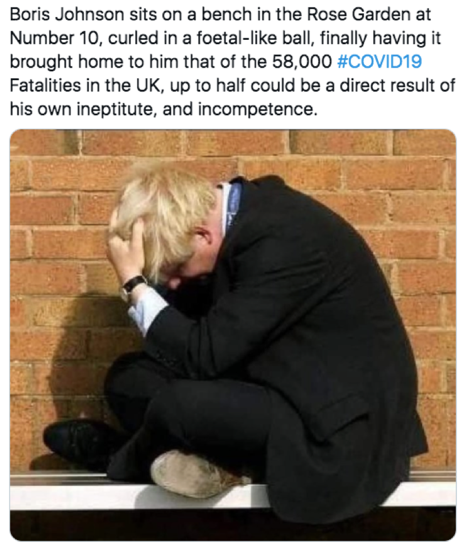

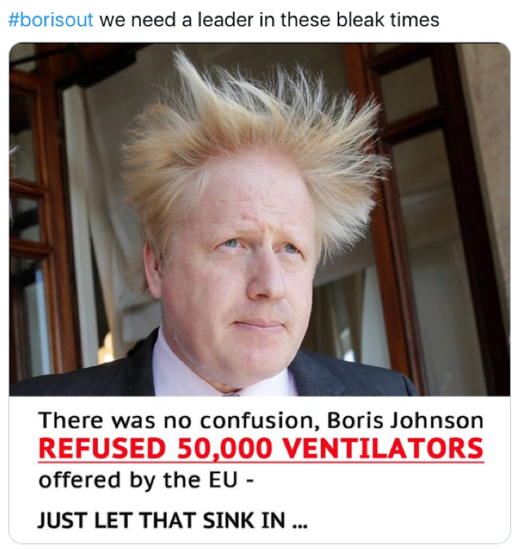

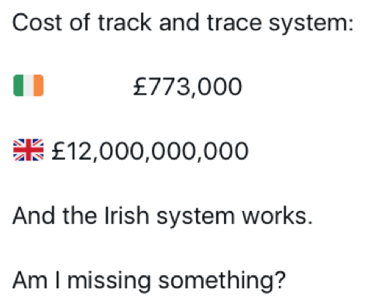


‘Favourable’ towards the UK Government (Study 1 & 2)


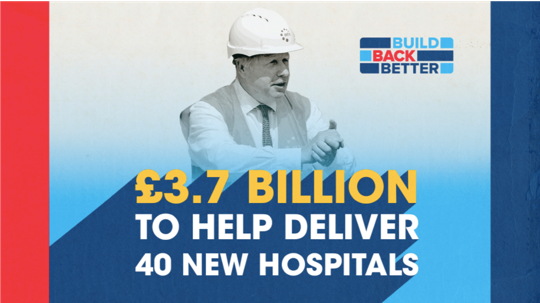

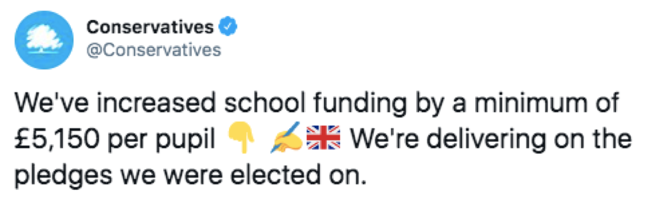

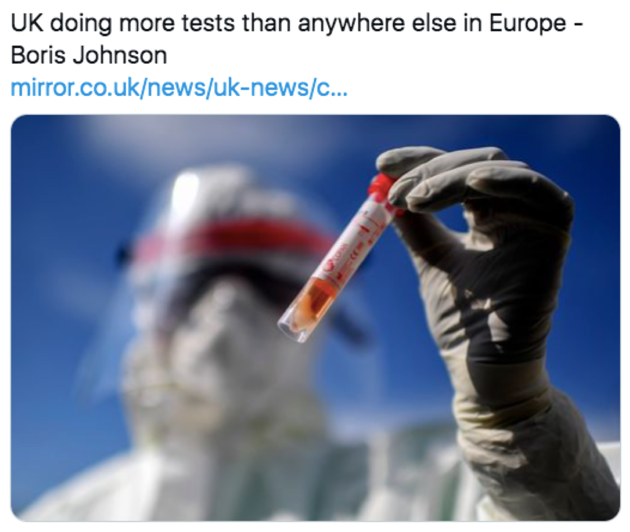


‘Minimising’ risk of COVID-19 (Study 1)


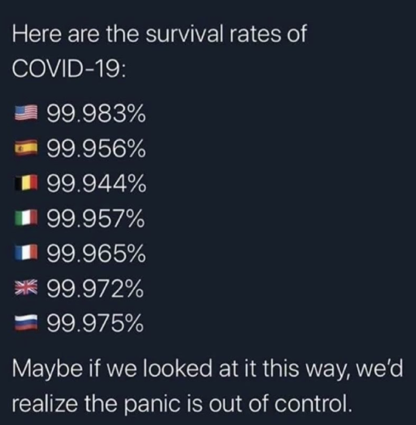

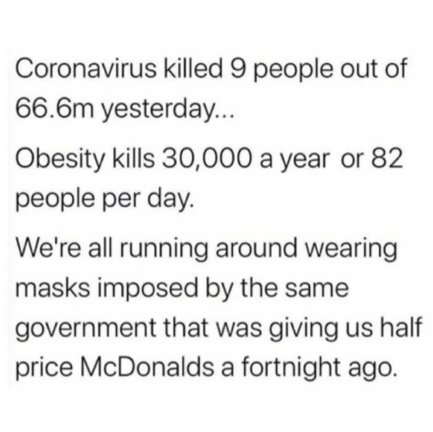

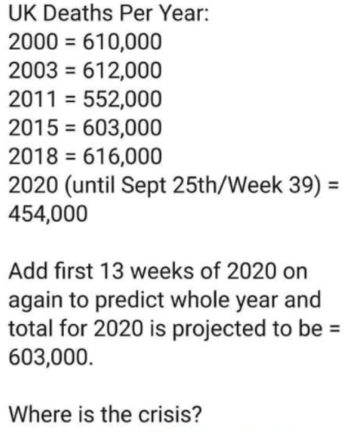


‘Maximising’ the risk of COVID-19 (Study 1)


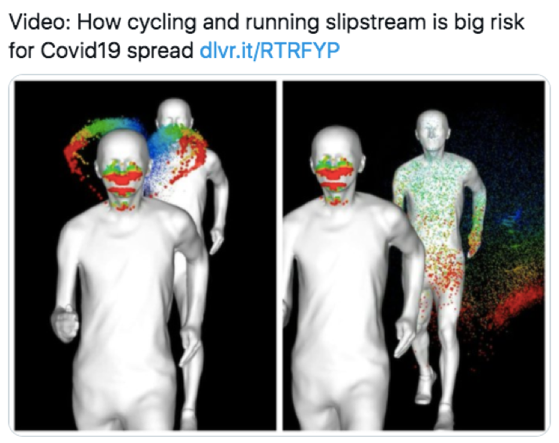

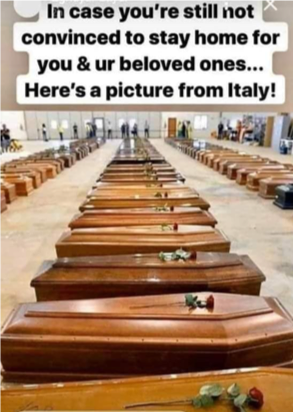

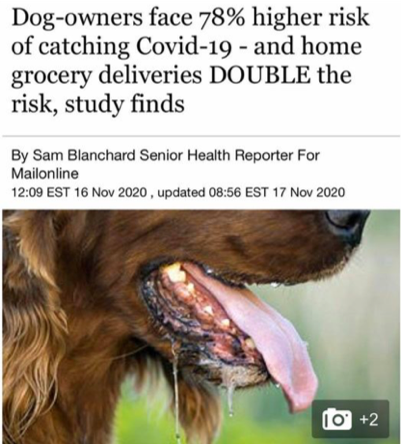

Supplement: S2 File — (DOCX) [file pone.0281777.s002.docx]
