# Supplementary material for: Moral leniency towards belief-consistent disinformation may help explain its spread on social media
Source: PLoS One. 2023 Mar 22;18(3):e0281777. doi: 10.1371/journal.pone.0281777 (PMC10032519; doi:10.1371/journal.pone.0281777)
Supplement: S3 File — (DOCX) [file pone.0281777.s003.docx]

S3. Supporting tables for Studies 1 and 2.

Study 1.

**S3A. Summary of Simple Slopes of Trust in Government for Likelihood of Interacting with Misinformation (Study 1)**

|  |  |  | *95% Confidence Interval* | |  |
| --- | --- | --- | --- | --- | --- |
|  | *Estimate* | *SE* | *Lower* | *Upper* | *t* |
| Favourable | 0.20*** | 0.05 | 0.09 | 0.31 | 3.65 |
| Unfavourable | -0.29*** | 0.06 | -0.41 | -0.18 | -4.88 |
| Minimising | -0.00 | 0.06 | -0.12 | 0.12 | -0.05 |
| Maximising | 0.01 | 0.04 | -0.08 | 0.10 | 1.09 |

*Note.* **p* < .05. ** *p* < .01. *** *p* < .001.

**S3B. Summary of Simple Slopes of Perceived Risk for Likelihood of Interacting with Misinformation (Study 1)**

|  |  |  | *95% Confidence Interval* | |  |
| --- | --- | --- | --- | --- | --- |
|  | *Estimate* | *SE* | *Lower* | *Upper* | *t* |
| Favourable | 0.10 | 0.13 | -0.16 | 0.35 | 0.73 |
| Unfavourable | 0.13 | 0.14 | -0.15 | 0.42 | 0.93 |
| Minimising | -0.16 | 0.15 | -0.45 | 0.13 | -1.07 |
| Maximising | 0.38** | 0.12 | 0.14 | 0.62 | 3.14 |

*Note.* **p* < .05. ** *p* < .01. *** *p* < .001.

**S3C. Summary of Simple Slopes of Trust in Government for Moral Acceptability of Spreading Disinformation (Study 1)**

|  |  |  | *95% Confidence Interval* | |  |
| --- | --- | --- | --- | --- | --- |
|  | *Estimate* | *SE* | *Lower* | *Upper* | *t* |
| Favourable | 0.16* | 0.07 | 0.03 | 0.30 | 2.33 |
| Unfavourable | -0.30*** | 0.06 | -0.42 | -0.17 | -4.57 |
| Minimising | 0.04 | 0.06 | -0.08 | 0.17 | 0.68 |
| Maximising | -0.00 | 0.06 | -0.12 | 0.11 | -0.08 |

*Note.* **p* < .05. ** *p* < .01. *** *p* < .001.

**S3D. Summary of Simple Slopes of Perceived Risk for Moral Acceptability of Spreading Disinformation (Study 1)**

|  |  |  | *95% Confidence Interval* | |  |
| --- | --- | --- | --- | --- | --- |
|  | *Estimate* | *SE* | *Lower* | *Upper* | *t* |
| Favourable | -0.28 | 0.17 | -0.62 | 0.05 | -1.69 |
| Unfavourable | -0.39* | 0.16 | -0.70 | -0.09 | -2.53 |
| Minimising | -0.71*** | 0.16 | -1.03 | -0.39 | -4.36 |
| Maximising | -0.02 | 0.14 | -0.30 | 0.26 | -0.15 |

*Note.* **p* < .05. ** *p* < .01. *** *p* < .001.

Study 2.

**S3E. Summary of Multilevel Mediation with Standard Errors Predicting Intentions to Spread Favourable Misinformation (Study 2)**

|  |  | Outcome | | |
| --- | --- | --- | --- | --- |
|  |  | *M*: Moral Judgement |  | *Y*: Likelihood of Spread |
| Within-Effects |  |  |  |  |
| Constant |  | 1.44*** (0.28) |  | -0.32** (0.11) |
| M: Moral Judgement |  |  | *b_L1_* → | 0.16*** (0.02) |
| Between-Effects |  |  |  |  |
| X: Trust | *a* → | 0.49*** (0.12) | *c'* → | 0.13** (0.04) |
| M: Moral Judgement |  |  | *b_L2_* → | 0.12*** (0.02) |
| Age |  | -0.04** (0.01) |  | -0.01 (0.01) |
| Gender |  | -0.98 (0.35) |  | 0.12 (0.13) |
|  |  |  |  |  |
| Variance components |  |  |  |  |
| Residual Estimates |  | 2.23*** (0.14) |  | 0.52*** (0.03) |
| Random Effect Estimates |  | 5.77*** (0.59) |  | 0.72*** (0.08) |
|  |  |  |  |  |
| Additional Information |  |  |  |  |
| -2 log likelihood (-2LL) |  |  | 5235.30 |  |
| Number of estimated parameters |  |  | 14 |  |
|  |  |  | Index | 95% bootstrap CI^a^ |
| Between-Direct Effect |  |  | 0.13 | 0.04, 0.22 |
| Between-Indirect Effect |  |  | 0.06 | 0.02, 0.10 |

*Note*. Gender coded as a dummy variable (0 = male, 1 = female).

^a^ Percentile bootstrap CI based on 5,000 bootstrap samples.

**p* < .05. ** *p* < .01. *** *p* < .001.

**S3F. Summary of Multilevel Mediation with Standard Errors Predicting Intentions to Spread Unfavourable Misinformation (Study 2)**

|  |  | Outcome | | | |
| --- | --- | --- | --- | --- | --- |
|  |  | *M*: Moral Judgement |  | *Y*: Likelihood of Spread |  |
| Within-Effects |  |  |  |  |  |
| Constant |  | -0.73*** (0.17) |  | 0.19 (0.13) |  |
| M: Moral Judgement |  |  | *b_L1_* → | 0.18*** (0.02) |  |
| Between-Effects |  |  |  |  |  |
| X: Trust | *a* → | -0.73*** (0.12) | *c'* → | -0.12* (0.06) |  |
| M: Moral Judgement |  |  | *b_L2_* → | 0.26*** (0.03) |  |
| Age |  | -0.03* (0.01) |  | -0.001 (0.01) |  |
| Risk |  | 0.13 (0.24) |  | 0.28 (0.11) |  |
|  |  |  |  |  |  |
| Variance components |  |  |  |  |  |
| Residual Estimates |  | 3.39*** (0.21) |  | 0.81*** (0.05) |  |
| Random Effect Estimates |  | 5.74*** (0.62) |  | 1.11*** (0.13) |  |
|  |  |  |  |  |  |
| Additional Information |  |  |  |  |  |
| -2 log likelihood (-2LL) |  |  | 5928.94 |  |  |
| Number of estimated parameters |  |  | 14 |  |  |
|  |  |  | Index | 95% bootstrap CI^a^ |  |
| Between-Direct Effect |  |  | -0.12 | -0.23, -0.003 |  |
| Between-Indirect Effect |  |  | -0.19 | -0.27, -0.12 |  |

*Note*. ^a^ Percentile bootstrap CI based on 5,000 bootstrap samples.

**p* < .05. ** *p* < .01. *** *p* < .001.
