# Supplementary material for: Moral leniency towards belief-consistent disinformation may help explain its spread on social media
Source: PLoS One. 2023 Mar 22;18(3):e0281777. doi: 10.1371/journal.pone.0281777 (PMC10032519; doi:10.1371/journal.pone.0281777)
Supplement: S4 File — (DOCX) [file pone.0281777.s004.docx]

S4. Study 2 – Pre-registered analyses

A reviewer noted that participants had rated multiple items, and therefore suggested that multilevel analyses would be appropriate. These are presented in the main paper. While the overall outcome does not change, the analyses for study 2 were pre-registered and therefore the original analysis is presented here. Multiple regressions are presented for each misinformation category (predicting both intentions to spread and moral judgements). Two mediation analyses are then presented, where moral judgements mediate the relationship between belief and intentions to spread.

Results

To test whether the belief-consistency of misinformation increased the likelihood that social media users would contribute to its spread (H1), two separate multiple regressions were carried out. Those who had low trust in the government were more likely to spread misinformation unfavourable towards the government compared to those with high trust (Table S4A), *ß* = ‑.31, *t*(241) = -4.76, *p* < .001. High trust instead predicted greater likelihood than others of spreading favourable misinformation about the government (Table S4B), *ß* = .25, *t*(241) = 3.67, *p* < .001.

Table S4A. Multiple Regressions Predicting Likelihood of Spread for ‘Unfavourable’ Misinformation

|  | *B* | *SE B* | *β* | *t* | *p* | *95% C.I. for B* |
| --- | --- | --- | --- | --- | --- | --- |
| Model |  |  |  |  |  |  |
| Constant | 6.22*** | .45 |  | 13.86 | <.001 | [5.33, 7.10] |
| Age | ‑0.01 | .01 | ‑.09 | -1.35 | .18 | [-0.02, 0.004] |
| Gender ^a^ | 0.18 | .19 | .06 | 0.98 | .33 | [-0.18, 0.55] |
| Trust | ‑0.31*** | .06 | ‑.31 | -4.76 | <.001 | [-0.43, -0.18] |
| Risk | 0.30* | .13 | .14 | 2.37 | .02 | [0.05, 0.55] |
| *R^2^* |  | .15 |  |  |  |  |
| *Adjusted R^2^* |  | .14*** |  |  |  |  |
| *F* |  | 10.82 |  |  |  |  |

**p* < .05. ** *p* < .01. *** *p* < .001.

^a^ Male = 0, Female = 1. *N*=246 as 5 participants identifying as non-binary or not disclosing gender were excluded from this analysis.

Table S4B. Multiple Regressions Predicting Likelihood of Spread for ‘Favourable’ Misinformation

|  | *B* | *SE B* | *β* | *t* | *p* | *95% C.I. for B* |
| --- | --- | --- | --- | --- | --- | --- |
| Model |  |  |  |  |  |  |
| Constant | 5.18 | .38 |  | 13.54 | <.001 | [4.43, 5.94] |
| Age | ‑0.01* | .01 | ‑.16 | ‑2.34 | .02 | [‑0.03, -0.002] |
| Gender ^a^ | ‑0.16 | .16 | ‑.06 | ‑0.99 | .32 | [-0.47, 0.16] |
| Trust | 0.20*** | .06 | .25 | 3.67 | <.001 | [0.09, 0.31] |
| Risk | ‑0.02 | .11 | ‑.01 | ‑0.18 | .86 | [-0.24, 0.20] |
| *R^2^* |  | .06 |  |  |  |  |
| *Adjusted R^2^* |  | .05** |  |  |  |  |
| *F* |  | 4.15 |  |  |  |  |

**p* < .05. ** *p* < .01. *** *p* < .001.

^a^ Male = 0, Female = 1. *N*=246 as 5 participants identifying as non-binary or not disclosing gender were excluded from this analysis.

Finally, a series of multiple regressions suggest that belief-consistency also predicts moral judgements of spreading misinformation, prior to learning that the content is false or misleading. ‘Unfavourable’ misinformation was judged to be more morally acceptable to spread by those with corresponding beliefs of low trust than others (Table S4C), *ß* = ‑.37, *t*(241) = -5.91, *p* < .001. Spreading favourable misinformation was viewed as more morally acceptable by those with high trust compared to those with low trust (Table S4D), *ß* = .27, *t*(241) = 4.13, *p* < .001.

Table S4C. Multiple Regressions Predicting Moral Judgements of ‘Unfavourable’ Misinformation

|  | *B* | *SE B* | *β* | *t* | *p* | *95% C.I. for B* |
| --- | --- | --- | --- | --- | --- | --- |
| Model |  |  |  |  |  |  |
| Constant | 8.79*** | .87 |  | 10.13 | <.001 | [7.08, 10.49] |
| Age | ‑0.03* | .01 | ‑.15 | ‑2.44 | .02 | [-0.06, -0.01] |
| Gender ^a^ | ‑0.07 | .36 | ‑.01 | ‑0.21 | .84 | [-0.78, 0.64] |
| Trust | ‑0.73*** | .12 | ‑.37 | -5.91 | <.001 | [-0.98, -0.49] |
| Risk | 0.13 | .25 | .03 | 0.54 | .59 | [-0.35, 0.62] |
| *R^2^* |  | .20 |  |  |  |  |
| *Adjusted R^2^* |  | .19*** |  |  |  |  |
| *F* |  | 14.92 |  |  |  |  |

**p* < .05. ** *p* < .01. *** *p* < .001.

^a^ Male = 0, Female = 1. *N*=246 as 5 participants identifying as non-binary or not disclosing gender were excluded from this analysis.

**Table S4D. Multiple Regressions Predicting Moral Judgements of ‘Favourable’ Misinformation**

|  | *B* | *SE B* | *β* | *t* | *p* | | *95% C.I. for B* | | |
| --- | --- | --- | --- | --- | --- | --- | --- | --- | --- |
| Model |  |  |  |  | |  | |  |  |
| Constant | 8.32*** | .85 |  | 9.77 | | <.001 | | [6.64, 10.00] |  |
| Age | ‑0.04** | .01 | ‑.19 | -2.91 | | .004 | | [-0.07, -0.01] |  |
| Gender ^a^ | ‑0.89* | .35 | ‑.16 | ‑2.52 | | .01 | | [-1.59, -0.20] |  |
| Trust | 0.50*** | .12 | .27 | 4.13 | | <.001 | | [0.26. 0.74] |  |
| Risk | ‑0.40 | .24 | ‑.10 | ‑1.66 | | .10 | | [-0.88. 0.08] |  |
| *R^2^* |  | .12 |  |  | |  | |  |  |
| *Adjusted R^2^* |  | .11*** |  |  | |  | |  |  |
| *F* |  | 8.34 |  |  | |  | |  |  |

**p* < .05. ** *p* < .01. *** *p* < .001.

^a^ Male = 0, Female = 1. *N*=246 as 5 participants identifying as non-binary or not disclosing gender were excluded from this analysis.

Mediation analyses were carried out using the PROCESS macro (model 4) to test whether moral judgements of spreading the misinformation (which they did not know at that stage was untrue) mediated the relationship between belief-consistency and spread (H3). The first model (Table S4E) predicted likelihood of spreading ‘Unfavourable’ misinformation while controlling for age and perceived risk of COVID-19 (both significant predictors themselves in the previous regressions). Those with low trust in the Government’s handling of the pandemic were more morally accepting of spreading belief-consistent (e.g. ‘Unfavourable’) misinformation (*a* = ‑.37, t(247) = -5.98, *p* < .001) and these moral judgements were subsequently related to a higher likelihood of spread (*b* = .52, *t*(246) = 8.26, *p* < .001). Based on 5000 bootstrapped samples, this indirect effect (*ab* = ‑.19) was significantly different from zero (95% *CI* = [ ‑.27, ‑.12]), but only partially mediated the relationship between consistent belief and spread (*c’* = ‑.12, *t*(246) = -2.17, *p* < .05).

**Table S4E. Model Coefficients for ‘Unfavourable’ Misinformation Mediation Model**

|  |  | Consequent | | | | | | |
| --- | --- | --- | --- | --- | --- | --- | --- | --- |
|  |  | *M* (MORAL JUDGEMENT) | | |  | *Y* (SPREAD) | | |
| Antecedent |  | Coeff. | *SE* | *Beta* |  | Coeff. | *SE* | *Beta* |
| *X* (TRUST) | *a* | -0.73*** | .12 | -.37 | *c’* | -0.12* | .06 | -.12 |
| *M* (MORAL JUDGEMENT) |  |  |  |  | *b* | 0.26*** | .03 | .52 |
| C_1_ (RISK) | *f_1_* | 0.13 | .24 | .03 | *g_1_* | 0.28** | .11 | .14 |
| C_2_ (AGE) | *f_2_* | -0.03* | .02 | -.15 | *g_2_* | -0.001 | .01 | -.01 |
| Constant | *i_M_* | 8.75*** | .84 |  | *i_Y_* | 4.03*** | .45 |  |
|  |  |  |  |  |  |  |  |  |
|  |  | *R^2^* = 0.2 | | |  | *R^2^* = 0.36 | | |
|  |  | *F* (3, 247) = 20.37, *p* < .001 | | |  | *F* (4, 246) = 35.35, *p* < .001 | | |

**p* < .05. ** *p* < .01. *** *p* < .001.

The second model predicted likelihood of spreading ‘Favourable’ misinformation, controlling for age and gender (Table S4F). High trust was related to increased moral acceptance of spreading belief-consistent (e.g. ‘Favourable’) misinformation (*a* = .26, *t*(242) = 3.62, *p* < .001). This judgement was then related to an increased likelihood of spreading ‘Favourable’ misinformation (*b* = .37, *t*(241) = 5.94, *p* < .001). Again, there was an indirect effect (*ab* = .1) which was significantly different from zero based on 5000 bootstrapped samples (95% *CI* = [.04, .16]). This again partially mediated the relationship between consistent beliefs and contribution to misinformation spread (*c’* = .15, *t*(241) = 2.54, *p* < .05).

**Table S4F. Model Coefficients for ‘Favourable’ Misinformation Mediation Model**

|  |  | Consequent | | | | | | |
| --- | --- | --- | --- | --- | --- | --- | --- | --- |
|  |  | *M* (MORAL JUDGEMENT) | | |  | *Y* (SPREAD) | | |
| Antecedent |  | Coeff. | *SE* | *Beta* |  | Coeff. | *SE* | *Beta* |
| *X* (TRUST) | *a* | 0.48*** | .12 | .26 | *c’* | 0.13* | .05 | -.15 |
| *M* (MORAL JUDGEMENT) |  |  |  |  | *b* | 0.16*** | .03 | .37 |
| C_1_ (AGE) | *f_1_* | -0.04** | .01 | -.21 | *g_1_* | -0.01 | .01 | -.08 |
| C_2_ (GENDER) | *f_2_* | -0.98** | .35 | -.17 | *g_2_* | -0.003 | .15 | -.001 |
| Constant | *i_M_* | 7.48*** | .58 |  | *i_Y_* | 3.90*** | .33 |  |
|  |  |  |  |  |  |  |  |  |
|  |  | *R^2^* = 0.11 | | |  | *R^2^* = 0.18 | | |
|  |  | *F* (3, 242) = 9.57, *p* < .001 | | |  | *F* (4, 241) = 13.61, *p* < .001 | | |

*Note.* Gender coded as dummy variable, M = 0 , F = 1

**p* < .05. ** *p* < .01. *** *p* < .001.
